# Sex differences in the genetic architecture of depression

**DOI:** 10.1038/s41598-020-66672-9

**Published:** 2020-06-18

**Authors:** Hee-Ju Kang, Yoomi Park, Kyung-Hun Yoo, Ki-Tae Kim, Eun-Song Kim, Ju-Wan Kim, Sung-Wan Kim, Il-Seon Shin, Jin-Sang Yoon, Ju Han Kim, Jae-Min Kim

**Affiliations:** 1 Departments of Psychiatry, Chonnam National University Medical School, Gwangju, Korea; 2 Seoul National University Biomedical Informatics (SNUBI), Division of Biomedical Informatics, Seoul National University College of Medicine, Seoul, Korea

**Keywords:** Genomic analysis, Diagnostic markers, Depression

## Abstract

The prevalence and clinical characteristics of depressive disorders differ between women and men; however, the genetic contribution to sex differences in depressive disorders has not been elucidated. To evaluate sex-specific differences in the genetic architecture of depression, whole exome sequencing of samples from 1000 patients (70.7% female) with depressive disorder was conducted. Control data from healthy individuals with no psychiatric disorder (n = 72, 26.4% female) and East-Asian subpopulation 1000 Genome Project data (n = 207, 50.7% female) were included. The genetic variation between men and women was directly compared using both qualitative and quantitative research designs. Qualitative analysis identified five genetic markers potentially associated with increased risk of depressive disorder in females, including three variants (rs201432982 within *PDE4A*, and rs62640397 and rs79442975 within *FDX1L*) mapping to chromosome 19p13.2 and two novel variants (rs820182 and rs820148) within *MYO15B* at the chromosome 17p25.1 locus. Depressed patients homozygous for these variants showed more severe depressive symptoms and higher suicidality than those who were not homozygotes (i.e., heterozygotes and homozygotes for the non-associated allele). Quantitative analysis demonstrated that the genetic burden of protein-truncating and deleterious variants was higher in males than females, even after permutation testing. Our study provides novel genetic evidence that the higher prevalence of depressive disorders in women may be attributable to inherited variants.

## Introduction

Depressive disorders are a leading cause of global disease burden^1^. Epidemiological studies have consistently shown that depressive disorders are more prevalent among females throughout the world including Asian countries, occurring approximately two or three times more frequently in women than in men although the difference is smaller in Asian countries^2,3^. Moreover, the clinical characteristics and treatment outcomes of depressive disorders in women are different from those in men^3,4^. Much effort has been expended to determine the mechanisms underlying the sex differences observed in depressive disorders; however, no definite mechanisms have been reported.

The impact of sex on the heritability of depressive disorders has been explored based on the substantial genetic contributions (35–40%)^5^, although definite conclusions have not been reached. Previous linkage analyses^6,7^ and genome-wide association studies (GWAS)^8–12^ have reported sex-specific genetic associations, although the results of such studies have rarely been replicated, with the exception of an association with *PCLO*^8,9^. Moreover, some studies have reported that genetic influences on depressive disorders are stronger in males^13,14^, while others have found more pronounced genetic influences in females^15,16^, or reported no detectable differences in genetic effects between the sexes^10,11,17–19^. These discrepancies may be attributable to age differences, dissimilar diagnostic approaches or limitations in the methods used to detect genetic differences related to depression between the sexes.

An alternative genetic study design that can compensate for the pitfalls of previous genetic studies is needed to identify the sex differences in the genetic contributions to depressive disorders. In turn, this would increase understanding of the pathogenesis of depressive disorders and lead to the development of new therapeutic targets. Large-scale whole exome sequencing (WES) data, combined with sufficient clinical information, have the potential to address the limitations of previous studies regarding sex differences in genetic effects on depressive disorders. Previous GWAS have been successful in identifying indirect genetic associations that could potentially contribute to depressive disorders; however, those common, low-impact genetic variations are insufficient to explain the entire genetic background of depressive disorders, even using meta-analytic methods to increase statistical power^20,21^. Additionally, previous GWAS results are not generalizable, due to an overwhelming bias towards the study of European populations^22^. To find the missing heritability of depressive disorders, recent studies have applied WES^23–25^, which has the advantage of being able to capture the mutual interplay between common and rare genetic variations in protein-coding regions; however, sex difference in genetic liabilities for depressive disorders have never been investigated using large-scale WES data. In this study, we used WES data from 1000 Korean patients with depressive disorder, along with highly curated clinical information, to investigate differential genetic effects between the sexes, based on both the effects of single variants and the cumulative effects of both rare and common variants. Additionally, to overcome the bias of case-control groups (groups effects with disease-vulnerable depressive patients from a hospital setting and disease-free healthy population from a community setting rather than pure sex-specific effect), direct comparisons were made between men and women within the depressive population and healthy control population separately, under the assumption that the net mutation rate does not differ between the sexes^26^. This approach is in in contrast to the traditional approach that compares cases with controls of the same sex.

Using this approach, we aimed to evaluate genetic effects according to sex status on depressive disorders, particularly in terms of prevalence, using WES data from 1000 Korean patients with depressive disorder.

## Methods

### Participants and datasets

The depressive disorders dataset was from the MAKE Biomarker discovery for Enhancing Antidepressant Treatment Effect and Response (MAKE BETTER) study, the details of which were published previously^27^. The MAKE BETTER study was designed to investigate biomarkers for treatment response, using a prospective design; the present study used baseline data from all participants who agreed to provide blood samples for genetic testing. Patients with major depressive disorders, dysthymic disorders, and depressive disorders not otherwise specified, who had recently visited the psychiatric department of Chonnam National University Hospital, were consecutively recruited. Detailed eligibility criteria are described in the Supplementary Methods. Depressive disorders were evaluated by study psychiatrists using the Mini-International Neuropsychiatric Interview (MINI), a structured diagnostic psychiatric interview based on the Diagnostic and Statistical Manual of Mental Disorders, Fourth Edition (DSM-IV) criteria^28^.

Two control group datasets were used in the present study. The first was a subsample from the Biomarker-Based Diagnostic Algorithm for Posttraumatic Syndrome (BioPTS) study, which investigated biomarkers predicting post-traumatic syndrome, including depression, anxiety, or post-traumatic stress disorder (PTSD), in a 2 year prospective study of patients with severe traumatic physical injury^29^. Among 141 participants in the BioPTS study, 72 who had no psychiatric disorders (i.e., depression, anxiety, or post-traumatic stress disorders) during the 2 year follow-up, even after severe physical injury, and who agreed to provide blood samples for genetic testing, were included as a control group in the present analyses. Absence of psychiatric disorders was defined by the study psychiatrists using MINI^28^ and the Clinician-Administered PTSD Scale-5 (CAPS-5)^30^, modified by DSM-5 criteria^31^. The detailed study protocols were described previously^29^, and the eligibility criteria of BioPTS are summarized in the Supplementary Methods. The second control dataset was obtained from the 1000 Genomes Project, Phase 3 (1KGP) public database^32^, and comprised data from individuals who declared themselves to be healthy, without any specific clinical phenotype, at the time of sample collection. Of 26 subpopulations in the 1KGP, the Han Chinese in Beijing, China (CHB), and the Japanese in Tokyo, Japan (JPT), who are located within a fairly narrow geographical range and genetically similar to the Korean population^33^, were used as an additional control group in the present investigation.

All participants of the MAKE BETTER study and BioPTS provided written informed consent. Both studies were conducted in accordance with institutional guidelines and the 1964 Declaration of Helsinki and were approved by the Chonnam National University Hospital Institutional Review Board.

### Whole exome sequencing

DNA was extracted from venous blood samples from participants of the MAKE BETTER and BioPTS studies who consented to genetic testing. WES was performed to screen coding sequence regions across the entire genome, using the Illumina HiSeq. 2500 sequencer (Illumina, Inc., San Diego, CA) with a standard protocol, as described in the manufacturer’s instructions. Detailed analysis procedures are described in the Supplementary Methods.

### Demographic and clinical characteristics of depressive patients

Demographic and clinical characteristics potentially associated with depressive disorder in men and women were considered in the present analyses. Demographic data included age, years of education, employment status, and number of chronic physical disorders. Clinical characteristics of depressive disorder were also evaluated as follows: Diagnosis of depressive disorders; number of depressive episodes; age of onset; duration of current episode; family history of depressive disorder; past history of suicide attempt; severity of depressive symptoms, according to the Hamilton Rating Scale for Depression^34^; severity of anxiety symptoms, according to the anxiety subscale of the Hospital Anxiety Depression Scale^35^; severity of suicidal ideation, according to the suicide-related items on the Brief Psychiatric Rating Scale^36^; and specific depression subtypes, including melancholic, atypical, and psychotic features, based on the DSM-IV criteria^37^.

### Statistical analyses

Analyses were designed to compare the influence of genetic effects on depressive disorder between the sexes, particularly in terms of prevalence, using qualitative and quantitative analytic design, as described in previous studies of other psychiatric disorders presenting sex-biased prevalence^38.39^. The qualitative analytic design was based on the qualitative hypothesis that certain variants contributed differentially to depressive disorders in a sex-specific manner. The quantitative analytic design reflected the quantitative hypothesis that the preponderance of depressive disorders in females might be attributable to a protective effect in males, whereby male individuals require a higher burden of genetic liability to develop depressive disorders.

To estimate genetic effects according to sex status in the qualitative analysis design, the analysis scheme summarized in Supplementary Fig. S1 was employed. Variant frequencies for all functional variants (missense, stop gained, stop lost, and start lost categories) were compared between men and women using Fisher’s exact test. Under the assumption that the net mutation rate between males and females in healthy controls does not differ significantly^26^, the minimum P-value obtained in the control data (P = 5.12E-05) was regarded as the empirical threshold, indicating the minimal clinically important difference between the male and female groups. To assess bias, the statistical significance of differences between the sexes among depressive patients was compared with that for control groups (psychiatric disease-free controls after severe injury, and the combined 1KGP CHB and JPT populations). In the depressive disorder group, variants below the threshold were considered to be significantly associated with sex-specific genetic architecture. Single-variant analysis was conducted using Fisher’s exact test based on allele frequencies. Three additional association tests including Fisher’s exact test based on genotype frequencies under both dominant and recessive models, and the Cochran-Armitage trend test were also performed to test the robustness of the results. The independent effects of variants on depressive disorder in the two sexes were estimated using a multivariate logistic regression model and the Cochran-Mantel-Haenszel test, after adjustment for potentially significant demographic and clinical characteristics (P < 0.1; broader significance cut-off to allow for “negative” confounding) identified by comparisons between men and women with depressive disorders, using *t*-, χ^2^, or Fisher’s exact tests, as appropriate. To evaluate the clinical impact of sex-specific variants in both the total population with depressive disorder and men and women separately, the clinical characteristics of depressive patients homozygous for associated variants carriers were compared with those of heterozygotes or non-carriers, using the Wilcoxon rank-sum or chi-squared test, as appropriate. Additionally haplotype analyses were performed for the five identified variants. Haplotypes were inferred using PHASE v.2.0^40^. The clinical characteristics of depressive patients carrying the haplotype consisting of five alternative variants in both alleles (homozygous) were compared with those of heterozygotes or non-carriers using the Wilcoxon rank-sum test. Consistent with previous studies^10,35,41^, the severity of depressive disorder was defined by age at onset, recurrence episode frequency, family history of depressive disorder, general severity according to baseline Hamilton Rating Scale for Depression scores, higher suicidality according to suicidal items from the Brief Psychiatric Rating Scale (≥ 4), and comorbid anxiety symptoms according to Hospital Anxiety Depression Scale anxiety subscale scores. Bonferroni corrections were used to correct an overall type I error rate of 0.05 against multiple comparisons, namely seven comparisons (0.05/7 = 0.007) in the analyses of clinical effects.

To estimate genetic effects according to sex status in the quantitative analysis design, variants were annotated and grouped into three annotation categories (Supplementary Table S1): i) Allele frequencies (‘Rare’ if minor allele frequency in the 1KGP < 0.1% or any other variants); ii) Functionality (‘Functional’ if included among missense, splice site, frameshift, stop lost, stop gain, stop retained, start lost, or in-frame InDels, or ‘Protein-truncating variants (PTVs)’ if included in splice site, frameshift, stop lost, stop gain, stop retained, or start lost); and iii) Deleteriousness (‘Deleterious’ if SIFT ≤ 0.05 or CADD ≥ 15 or any other variants). Using these annotation categories, eight category combinations (Rare functional, Functional, Rare functional deleterious, Functional deleterious, Rare PTVs, PTVs, Rare PTVs deleterious, and PTVs deleterious) were created for both 17,512 autosomal protein-coding genes with ≥ 1 variant and 967 autosomal genes previously reported to have genetic associations with MDD in the MK4MDD database^42^. For each category combination, the genetic burden of individual subjects was estimated by counting the number of variants or genes overlapping the defined variant categories, and comparing the results between men and women using the Wilcoxon rank-sum test. To test how often the model would arise by chance using patient data with randomly permuted sex labels, permutation tests were conducted as follows (Supplementary Fig. S2): (i) Under the null hypothesis (i.e., that there is no significant difference in variant or gene burden between males and females), the sex labels of patient data were permuted, resulting in the same numbers as those in non-permuted data (i.e., 707 females and 293 males) of assigned males and females in a random order; ii) For this permuted dataset, the genetic burden between data assigned ‘male’ and ‘female’ was compared using the Wilcoxon rank-sum test; iii) The test statistic ‘*W*’ obtained using the Wilcoxon rank-sum test was recorded; iv) Steps i) through iii) were repeated 10,000 times (*T* = 10,000). The permuted P-value (*C*/*T*) was calculated by counting how many times *W* values from analysis of the permuted datasets were larger than the *W* value obtained using the observed dataset (*C*). In order to make comparisons using Polygenic risk scores (PRSs), PRSs were constructed via PLINK^43^ and PRSice-2^44^ with SNP weights based on recent PGC-MDD summary statistics of the European population (https://www.med.unc.edu/pgc/download-results/mdd/)^45^. Common SNPs (MAF > 0.01) identified in the 1KGP European population were clumped using LD parameters of r^2^ > 0.1 in a 250 kb window. PRSs were obtained using LD-clumped independent SNPs with p-values for association below eight thresholds (P < 10^−4^, 0.001, 0.01, 0.05, 0.1, 0.2, 0.5 and 1). PRSs between men and women were compared using the Wilcoxon rank-sum test in depressive patients and in healthy controls from the BioPTS study.

All statistical analyses were conducted using R (v3.2.3; http://www.r-project.org/).

## Results

### Subject description

All samples included in the analyses are summarized in Supplementary Table S2. Among 1265 depressive patients who participated in the MAKE BETTER study, 1000 consented to blood sampling for genotyping and these comprised the depressive disorders sample in the present investigation. The baseline characteristics of those who did and did not consent to blood sampling did not differ significantly (all P > 0.066), excluding the number of depressive episodes, the severity of suicidal ideation, and melancholic features. Patients who refused to provide blood samples were likely to have more depressive episodes (P < 0.001), were less likely to be unemployed (P = 0.003), and had less severe suicidal ideation (P = 0.039), and more melancholic features (P = 0.037). Of the 1000 included patients with depressive disorders, 707 (70.7%) were female. The characteristics of female and male depressive patients are described in Table 1. Female patients were more likely to have a lower education level, be unemployed, and experience recurrent depressive episodes, and have more severe depressive symptoms.

**Table 1:** Demographic and clinical characteristics of depressive patients.

Variables	Females (N = 707)	Males (N = 293)		P-value^*^
**Socio-demographic characteristics**				
Age, mean(SD) years	57.2 (13.8)	55.9 (16.3)	*t* = −1.18	0.237
Education, mean(SD) years	8.3 (4.8)	11.0 (4.3)	*t* = −8.71	**3.13 E-17**
Marital status, n(%), married	492 (69.6)	209 (71.3)	χ^2^ = 0.22	0.637
Unemployment status, n(%)	171 (24.2)	101 (34.5)	χ^2^ = 10.55	**0.001**
**Clinical characteristics**				
Diagnosis, n(%)				
Major depressive disorder	614 (86.8)	241 (82.3)	χ^2^ = 3.16	**0.075**
Other depressive disorder	93 (13.2)	52 (17.7)		
Episode of depression				
Recurrent depression (yes), n(%)	382 (54.0)	128 (43.7)	χ^2^ = 8.46	**0.003**
Number of depression episode	1.9 (4.2)	1.6 (4.2)	*t* = 1.12	0.263
Age at onset, mean(SD) years	51.6 (15.8)	52.9 (17.7)	*t* = −1.12	0.264
Duration of illness, mean(SD) day	284.0 (658.3)	307.5 (686.8)	*t* = −0.50	0.618
Family history of depression (yes), n(%)	116 (16.4)	34 (11.6)	χ^2^ = 3.38	**0.066**
History of suicide attempt (yes), n(%)	55(7.8)	31(10.6)	χ^2^ = 1.73	0.189
Assessment scales, mean(SD) scores				
Hamilton Depression Rating Scale	20.9 (4.3)	20.2 (4.1)	*t* = 2.61	**0.009**
Anxiety-subscale of Hospital Anxiety and Depression Scale	11.7 (4.0)	11.9 (4.0)	*t* = −0.46	0.646
Suicide item of Brief Psychiatric Rating scale				
Mild or less(1–3), n(%)	468 (66.2)	199 (67.9)	χ^2^ = 0.20	0.651
Moderate to extreme severe(4–7), n(%)	239(33.8)	94(32.1)		
Features of depression, n(%)				
atypical feature (yes)	51 (7.2)	17 (5.8)	χ^2^ = 0.45	0.504
melancholic feature (yes)	97 (13.7)	45 (15.4)	χ^2^ = 0.33	0.565
psychotic feature (yes)	44 (6.2)	12 (4.1)	χ^2^ = 1.39	0.238

^*^t-test, χ^2^ test, or Fisher’s exact tests, as appropriate.

Values in bold type show broader significance cut-off (P < 0.1).

The first control dataset comprised 72 patients from the BioPTS who had not experienced any psychiatric disorders during 2 year follow-up, even after severe physical injury. In contrast to the depression group, there were more males (73.6%) than females (26.4%) in the BioPTS group. The second control dataset included CHB and JPT subpopulation data (n = 207) from the 1KGP (n = 2504). Approximately half (50.7%) of the combined CHB and JPT group was female.

### Sex-specific genetic heterogeneity associated with depression determined by qualitative analyses

Frequency distributions of the 236,274 functional variants identified in the 1000 patients with depressive disorders were compared between men and women using Fisher’s exact test (Supplementary Fig. S1). Five variants were found to have clinically important significant differences between men and women with depressive disorder (Table 2; P < 5.12E-05) and these were almost twice as frequent in females as males in this group; however, none of the five variants differed in frequency in either control group. Three of the five, which mapped to the phosphodiesterase 4A (*PDE4A*) (rs201432982) and ferredoxin 1 like (*FDX1L*) (rs62640397 or rs79442975) loci, were in the same chromosomal cytoband (19p13.2), while the other two variants (rs820182 and rs820148), which were in Myosin-XVB (*MYO15B*), were on chromosome 17p25.1. The two variants in the *FDX1L* gene were in complete linkage disequilibrium (D’ = 1) in the entire 1KGP subpopulation. Odd ratios calculated using both dominant and recessive model analysis for all five variants indicated that they were more frequent among depressive females than males with this disorder. Further potential candidate genes, with marginal P-values (P < 1E-03), are summarized in Supplementary Table S3. The independent effects of the variants on depressive disorders by sex status, determined using a multivariate logistic regression model and the Cochran-Mantel-Haenszel test after adjustment for potential demographic and clinical characteristics, are summarized in Supplementary Table S4. Even after adjustment, the P-values remained around the suggestive level of significance (suggestive P = 1E-04 and P = 2E-02, respectively).

**Table 2:** Sex-specific variants detected in depressive disorder compared with those in the general population.

Gene	Variant/position	SIFT	CADD	PP2	Exon	HGVS.p	Type	Group	Sex	Allele frequency			Genotype frequency							
										MAF	Fisher's p	OR (95% CI)	REF	HET	HOM	Dominant		Recessive		CATT p
																Fisher's p	OR (95% CI)	Fisher's p	OR (95% CI)	
*PDE4A*	rs201432982/19p13.2	0.04	23.5	NA	1/15	p.Arg57Trp	Missense	MDD, N=1000	Female	0.060	2.86E-05	2.6 (1.6-4.5)	598	98	11	1.68E-04	2.5 (1.5-4.3)	0.040	Inf (1.1-Inf)	0.0002
									Male	0.010			273	20	0					
								CHBJPT, N=207	Female	0.024	0.830	0.88 (0.33-2.3)	95	10	0	1	0.97 (0.34-2.7)	0.493	0 (0-38)	0.789
									Male	0.027			92	9	1					
								BioPTS, N=53	Female	0.014	0.654	1.4 (0.1-10)	17	2	0	0.651	1.4 (0.1-11)	1	0 (0-Inf)	0.459
									Male	0.028			49	4	0					
*FDX1L (RAVER1 downstream)*	rs62640397/19p13.2 rs79442975/19p13.2	0	0.017	0.069	1/5	p.Arg29Gly	Missense	MDD, N=1000	Female	0.073	3.69E-05	2.3 (1.5-3.6)	574	121	12	1.35E-04	2.3 (1.5-3.7)	0.123	5 (0.74-216)	0.0001
									Male	0.014			266	26	1					
		NA	9.108	NA	1/4	NA	Splice region variant	CHBJPT, N=207	Female	0.031	0.834	1.2 (0.47-2.9)	92	13	0	0.660	1.3 (0.5-3.5)	0.493	0 (0-38)	0.758
									Male	0.027			92	9	1					
								BioPTS, N=53	Female	0.007	0.445	0.33 (0-2.6)	18	1	0	0.429	0.32 (0-2.7)	1	0 (0-Inf)	0.615
									Male	0.056			45	8	0					
*MYO15B*	rs820182/ 17p25.1	NA	5.116	NA	48/62	NA	Splice region variant	MDD, N=1000	Female	0.137	1.47E-05	1.9 (1.4-2.5)	458	225	24	5.02E-05	1.9 (1.4-2.7)	0.014	5.1 (1.2-45)	1.63E-05
									Male	0.034			228	63	2					
								CHBJPT, N=207	Female	0.085	0.700	0.9 (0.52-1.6)	72	31	2	0.882	0.92 (0.49-1.7)	0.680	0.64 (0.05-5.7)	0.687
									Male	0.089			68	31	3					
								BioPTS, N=53	Female	0.035	1	1.00 (0.3-3.2)	14	5	0	1	1.1 (0.26-4.1)	1	0 (0-109)	0.805
									Male	0.097			40	12	1					
*MYO15B*	rs820148/17p25.1	NA	6.033	NA	45/62	NA	Splice region variant	MDD, N=1000	Female	0.125	4.67E-05	1.8 (1.3-2.5)	478	208	21	2.30E-04	1.8 (1.3-2.6)	0.008	8.9 (1.4-370)	5.85E-05
									Male	0.031			232	60	1					
								CHBJPT, N=207	Female	0.075	0.505	0.81 (0.46-1.4)	76	27	2	0.648	0.84 (0.44-1.6)	0.441	0.48 (0.04-3.4)	0.430
									Male	0.087			70	28	4					
								BioPTS, N=53	Female	0.028	1	0.84 (0.2-3.0)	15	4	0	1	0.91 (0.19-3.7)	1	0 (0-109)	0.986
									Male	0.090			41	11	1					

SIFT, Sorting Intolerant From Tolerant; PP2, = PolyPhen2; CADD, Combined annotation dependent depletion; HGVS.p, Human genome variation society –protein reference sequence; MAF, minor allele frequency; REF, reference allele; ALT, alternative allele; Fisher’s p, Fishers exact test p-value; OR, odd ratio; CATT p, Cochran-Armitage Trend Test p-value; MDD, major depressive disorder; CHBJPT, Han Chinese in Beijing and the Japanese in Tokyo, Japan; BioPTS, the Biomarker-Based Diagnostic Algorithm for Posttraumatic Syndrome Study; NA, not applicable.

To assess the clinical relevance of these variants, clinical characteristics indicating depression severity were compared between homozygotes for the associated SNPs and heterozygous or non-carriers, using the Wilcoxon rank-sum and chi-squared tests, as appropriate, under the hypothesis that two copies of the variants may lead to more serious symptoms (Table 3). Depressive patients homozygous for ≥ 1 of the variants exhibited more severe depressive symptoms, including higher baseline depressive scores and greater severity of suicidal ideation, even after Bonferroni correction. When the sample was split into males and females, only the female group showed associations with similar severe depressive symptoms even after Bonferroni correction, with improved statistical significance, while in males the clinical phenotypes of homozygous carriers and heterozygous or non-carriers did not differ significantly, excluding values that could not be calculated due to insufficient data. Each of the five variants showed similar trends, although the statistical significances remained only for higher baseline depressive scores in carriers of MYO15B after Bonferroni correction. Notably, in thirty-two depressive patients the haplotype consisting of five alternative variant (T-C-A for PDE4A-FDX1L and C-G for MYO15B) in both alleles (homozygous) was more prominent in female patients and was associated with similar clinical patterns (Supplementary Table S5), namely higher baseline depressive scores and greater severity of suicidal ideation. However, because of the limited number of variant carriers in the present study, future studies are needed to ascertain the associations between the five variants and their haplotypes and clinical outcomes. Nonetheless, our findings suggest that these five variants, which were more frequent in females with depression, could potentially influence sex-specific heterogeneity in the clinical features of depressive disorder.

**Table 3:** Clinical characteristics of depressive patients with and without sex-specific genetic variants.

Variables	Depressive patients, N = 1000				Depressive patients, Female, N = 707				Depressive patients, Male, N = 293			
**Total** **(5 variants)**	Carrier, N = 40	Non-carrier, N = 960	Statistical coefficient	P-value	Carrier, N = 37	Non-carrier, N = 670	Statistical coefficient	P-value	Carrier, N = 3	Non-carrier, N = 290	Statistical coefficient	P-value
HAMD baseline Score, mean (sd)	23.3 (4.5)	20.58 (4.2)	W = 25740.0	**2.48E-04**	23.35 (4.6)	20.78 (4.2)	W = 16277.0	**0.001**	22.67 (2.3)	20.13 (4.1)	W = 630.5	0.180
HADS-anxiety subscale, mean(sd)	12.7 (3.8)	11.7 (4.0)	W = 21808.0	0.144	12.9 (3.8)	11.7 (4.0)	W = 14568.0	0.072	10.0 (4.6)	11.9 (4.0)	W = 322.0	0.440
BPRS suicide item≥4, n(%)	24 (60.0)	309 (32.2)	*χ*2 = 12.15	**4.91E-04**	22 (59.5)	217 (32.4)	*χ*2 = 10.31	**0.001**	2 (66.7)	92 (31.7)	*χ*2 = 0.45	0.504
Suicidal attempt (yes), n(%)	5 (12.5)	81 (8.4)	*χ*2 = 0.37	0.542	5 (13.5)	50 (7.5)	*χ*2 = 1.05	0.307	0(0)	31 (10.7)	0	1
Family history (yes), n(%)	7 (17.5)	143 (14.9)	*χ*2 = 0.05	0.821	7 (18.9)	109 (16.3)	*χ*2 = 0.04	0.845	0(0)	34 (11.7)	0	1
Recurrent depression, n(%)	21 (52.5)	489 (50.9)	*χ*2 = 0	0.974	20 (54.1)	362 (54)	*χ*2 = 0	1	1 (33.3)	127 (43.8)	0	1
Age of onset, mean(sd)	50.1 (17.2)	52.0 (16.4)	W = 17712.0	0.144	50.7 (17.1)	51.6 (15.8)	W = 11879.0	0.670	42.7 (21.5)	53.0 (17.7)	W = 299.5	0.355
**PDE4A (rs201432982)**	Carrier, N = 11	Non-carrier, N = 989	Statistical coefficient	P-value	Carrier, N = 11	Non-carrier, N = 696	Statistical coefficient	P-value	Carrier, N = 0	Non-carrier, N = 293	Statistical coefficient	P-value
HAMD baseline Score, mean (sd)	22.91 (4.0)	20.67 (4.2)	W = 7132.0	0.075	22.91 (4.0)	20.88 (4.3)	W = 4915.0	0.105	NA	20.16 (4.1)	NA	NA
HADS-anxiety subscale, mean(sd)	12.2 (4.5)	11.8 (4.0)	W = 5820.0	0.689	12.2 (4.5)	11.7 (4.0)	W = 4108.5	0.676	NA	11.9 (4.0)	NA	NA
BPRS suicide item≥4, n(%)	7 (63.6)	326 (33.0)	*χ*2 = 3.33	0.068	7 (63.6)	232 (33.3)	*χ*2 = 3.19	0.074	0(0)	94 (32.1)	0	1
Suicidal attempt (yes), n(%)	1 (9.1)	85 (8.6)	*χ*2 = 0	1	1 (9.1)	54 (7.8)	*χ*2 = 0	1	0(0)	31 (10.6)	0	1
Family history (yes), n(%)	1 (9.1)	149 (15.1)	*χ*2 = 0.02	0.899	1 (9.1)	115 (16.5)	*χ*2 = 0.06	0.803	0(0)	34 (11.6)	0	1
Recurrent depression, n(%)	6 (54.5)	504 (51.0)	*χ*2 = 0	1	6 (54.5)	376 (54)	*χ*2 = 0	1	0(0)	128 (43.7)	0	1
Age of onset, mean(sd)	51.9 (15.2)	52.0 (16.4)	W = 5141.5	0.755	51.9 (15.2)	51.6 (15.9)	W = 3666.5	0.811	NA	52.9 (17.7)	NA	NA
**FDX1L (rs62640397/rs79442975)**	Carrier, N = 13	Non-carrier, N = 987	Statistical coefficient	P-value	Carrier, N = 12	Non-carrier, N = 695	Statistical coefficient	P-value	Carrier, N = 1	Non-carrier, N = 292	Statistical coefficient	P-value
HAMD baseline Score, mean (sd)	23.38 (3.5)	20.66 (4.2)	W = 9010.5	0.012	23.33 (3.7)	20.87 (4.3)	W = 5684.0	0.048	24.0 (NA)	20.15 (4.1)	W = 240.5	0.265
HADS-anxiety subscale, mean(sd)	12.2 (4.3)	11.8 (4.0)	W = 6829.5	0.689	12.4 (4.3)	11.7 (4.0)	W = 4644.5	0.498	9 (NA)	11.9 (4.0)	W = 75.0	0.403
BPRS suicide item≥4, n(%)	8 (61.5)	325 (32.9)	*χ*2 = 3.53	0.060	7 (58.3)	232 (33.4)	*χ*2 = 2.26	0.133	1 (100)	93 (31.8)	*χ*2 = 0.15	0.701
Suicidal attempt (yes), n(%)	2 (15.4)	84 (8.5)	*χ*2 = 0.14	0.704	2 (16.7)	53 (7.6)	*χ*2 = 0.38	0.538	0(0)	31 (10.6)	0	1
Family history (yes), n(%)	2 (15.4)	148 (15.0)	*χ*2 = 0	1	2 (16.7)	114 (16.4)	*χ*2 = 0	1	0(0)	34 (11.6)	0	1
Recurrent depression, n(%)	6 (46.2)	504 (51.1)	*χ*2 = 0.01	0.942	6 (50.0)	376 (54.1)	*χ*2 = 0	1	0(0)	128 (43.8)	0	1
Age of onset, mean(sd)	50.9 (20.4)	52.0 (16.4)	W = 6829.5	0.689	52.2 (20.7)	51.6 (15.8)	W = 4161.0	0.990	35(NA)	53.0 (17.7)	W = 51.5	0.266
**MYO15B (rs820182)**	Carrier, N = 26	Non-carrier, N = 974	Statistical coefficient	P-value	Carrier, N = 24	Non-carrier, N = 683	Statistical coefficient	P-value	Carrier, N = 2	Non-carrier, N = 291	Statistical coefficient	P-value
HAMD baseline Score, mean (sd)	23.42 (5.0)	20.62 (4.2)	W = 16729.5	**0.005**	23.54 (5.1)	20.82 (4.2)	W = 10665	0.018	22.0 (2.8)	20.15 (4.1)	W = 392.0	0.399
HADS-anxiety subscale, mean(sd)	13.0 (3.7)	11.7 (4.0)	W = 15016.5	0.104	13.3 (3.6)	11.7 (4.0)	W = 10009	0.065	10.5 (6.4)	11.9 (4.0)	W = 249.0	0.727
BPRS suicide item≥4, n(%)	15 (57.7)	318 (32.6)	*χ*2 = 6.07	0.014	140 (58.3)	225(32.9)	*χ*2 = 5.59	0.018	1 (50.5)	93 (32.0)	0	1
Suicidal attempt (yes), n(%)	3 (11.5)	83 (8.5)	*χ*2 = 0.04	0.852	3 (1.5)	52 (7.6)	*χ*2 = 0.24	0.624	0(0)	31 (10.7)	0	1
Family history (yes), n(%)	5 (19.2)	145 (14.9)	*χ*2 = 0.11	0.738	5 (20.8)	111 (16.3)	*χ*2 = 0.1	0.753	0(0)	34 (11.7)	0	1
Recurrent depression, n(%)	14 (53.8)	496 (50.9)	*χ*2 = 0.01	0.924	13 (54.2)	369 (54)	NA	NA	1 (50)	127 (43.6)	0	1
Age of onset, mean(sd)	50.0 (16.2)	52.0 (16.4)	W = 11784	0.546	50.3 (15.7)	51.6 (15.9)	W = 7830	0.710	46.5 (29)	53.0 (17.7)	W = 250.0	0.734
**MYO15B (rs820148)**	Carrier, N = 22	Non-carrier, N = 978	Statistical coefficient	P-value	Carrier, N = 21	Non-carrier, N = 686	Statistical coefficient	P-value	Carrier, N = 1	Non-carrier, N = 292	Statistical coefficient	P-value
HAMD baseline Score, mean (sd)	23.82 (5.3)	20.62 (4.2)	W = 14536.5	**0.005**	24.00 (5.3)	20.82 (4.2)	W = 9715.5	**0.006**	20.0 (NA)	20.16 (4.1)	W = 152.5	0.943
HADS-anxiety subscale, mean(sd)	13.0 (3.5)	11.7 (4.0)	W = 12743.5	0.137	13.3 (3.3)	11.7 (4.1)	W = 8940	0.059	6 (NA)	11.9 (4.0)	W = 24.0	0.15
BPRS suicide item≥4, n(%)	13 (59.1)	320 (32.7)	*χ*2 = 5.60	0.018	13 (61.9)	226 (32.9)	*χ*2 = 6.40	0.011	0(0)	94 (32.2)	0	1
Suicidal attempt (yes), n(%)	2 (9.1)	84 (8.6)	*χ*2 = 0	1	2 (9.5)	53 (7.7)	*χ*2 = 0	1	0(0)	31 (10.6)	0	1
Family history (yes), n(%)	5 (22.7)	145 (14.8)	*χ*2 = 0.52	0.469	5 (23.8)	111 (16.2)	*χ*2 = 0.4	0.528	0(0)	34 (11.6)	0	1
Recurrent depression, n(%)	11 (50)	499 (51)	*χ*2 = 0	1	11 (52.4)	371 (54.1)	*χ*2 = 0	1	0(0)	128 (43.8)	0	1
Age of onset, mean(sd)	50.3 (15.4)	52 (16.4)	W = 10063.0	0.604	49.5 (15.4)	51.6 (15.9)	W = 6653.5	0.551	67.0 (NA)	52.9 (17.7)	W = 221.0	0.378

P-Values were analyzed using the Wilcoxon rank-sum or chi-squared test, as appropriate.

Values in bold type show statistical significance after Bonferroni correction.

HAMD, Hamilton Depression Rating Scale; HADS, Hospital Anxiety Depression Scale; BPRS, Brief Psychiatric Rating Scale; NA, not applicable.

### Male protective effects in depression identified by quantitative analyses

**Figure 1:**
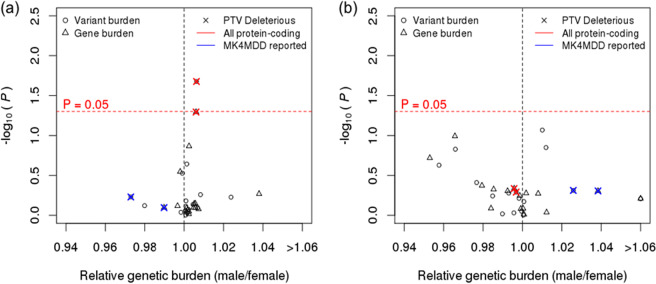
Comparison of the genetic burden between men and women in patients with depressive disorder and the general population. Comparison of genetic variants in each category (points) enriched in males (relative genetic burden > 1) or females (relative genetic burden < 1). The red dashed line indicates the significance threshold (0.05). (**a**) Patients with depressive disorder (n = 1000). (**b**) Healthy controls from the 1KGP CHB and JPT populations (n = 207).

The numbers of variants and genes overlapping eight defined annotation categories (Rare functional, Functional, Rare functional deleterious, Functional deleterious, Rare protein-truncating variants (PTVs), PTVs, Rare PTVs deleterious, and PTVs deleterious) were defined as the variant- and gene-level genetic burdens, respectively (see ‘Statistical analyses’ in the Supplementary Methods). Genetic burdens were compared between men and women for the genes previously associated with depression in the Multi-Level Knowledge Base and Analysis Platform for Major Depressive Disorder (MK4MDD) database^37^, and autosomal protein-coding genes, including as-yet-unidentified associations, using the Wilcoxon rank-sum test (Fig. 1). Among 32 annotation categories (8 categories × 2 (autosomal protein-coding genes and MK4MDD genes) × 2 (variant- and gene-level genetic burden), 78% (25/32) showed a higher genetic burden in males than females in the depressive disorders group, while random distributions were observed in healthy controls; 59.4% (19/32) showed higher genetic burdens in males. In the depressive disorders group, the variant-level genetic burden overlapping with deleterious PTVs in autosomal protein-coding genes was significantly higher in males (Fig. 1a; P = 0.021), while the gene-level genetic burden was marginally significant in the same category (P = 0.051). None of the categories reached statistical significance for genes reported in the MK4MDD database alone. In the healthy controls, there was no significant difference in the genetic burden between males and females, as expected (Fig. 1b). A similar trend was observed when the same analyses were conducted with the modified rare variant definition (allele frequency <1%; Supplementary Fig. S3).

**Figure 2:**
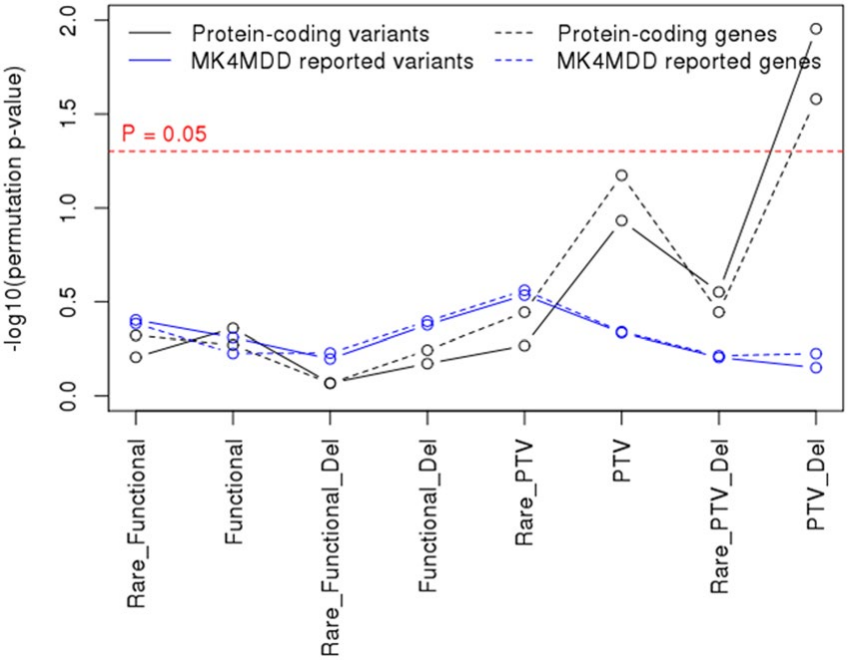
Results of permutation testing of differences in genetic burden according to sex. P-values of Wilcoxon rank-sum tests using 10,000 permutations of random male and female labels. Black and blue solid lines, depressive patients. Solid lines represent variant-level analyses, and dashed lines represent gene-level analyses.

To approximate the true distribution of the genetic burden, we conducted permutation tests by shuffling the sex labels for the dataset 10,000 times and comparing the genetic burden between the assigned sex labels using each of these randomly permuted sets. Deleterious PTVs remained significantly enriched in males following permutation analysis (Fig. 2; variant-level permutation, P = 0.011 and gene-level permutation, P = 0.026), indicating that the finding that male depressive disorders require a greater genetic burden is not due to random chance.

We further applied PRSs based on recent PGC-MDD summary statistics^45^ of the European population to validate male protective effects in depression using WES data (Supplementary Fig. S4). When the p-value threshold p < 0.01 was used, male patients with depressive disorder had significantly higher PRSs than female patients with depressive disorder (*p* = 0.045 by Wilcoxon rank-sum test). However, in the BioPTS control group, PRSs were not significantly different between men and women at any p-value threshold level.

## Discussion

The principal findings of the present genetic study using WES data are that five variants in the *PDE4A*, *FDX1L*, and *MYO15B* genes are associated with increased risk of depressive disorder in women, that depressive patients homozygous for these variants are more likely to experience severe depressive symptoms, and that a higher genetic burden is required for men to develop depressive disorder, particularly of protein-truncating and deleterious variants, which may contribute to the higher resilience of male individuals against depressive disorder. Overall, these findings provide evidence for genetic factors underlying the female preponderance in depressive disorder.

Recent studies have reported markedly different transcriptional patterns between males and females with depressive disorders^46^. To examine whether the increased prevalence of depressive disorders in females is associated with sex-specific molecular signatures related to genetic heterogeneity or liability, we applied a unique analytic strategy with several strengths, which directly compared genetic variation between males and females using WES data from Korean patients with depressive disorders. This large-scale WES analysis identified clinically meaningful common and rare coding variants, and enabled investigation of the evidence supporting genetic sex differences, using both single-variant and gene-level association tests. Moreover, the strategy of direct comparison between the sexes makes intuitive sense for understanding sex-related genetic differences associated with depressive disorders. This strategy also reduces the systemic bias introduced by the use of two different datasets (e.g., in-house and publicly available data), which were generated using differing data processing protocols. In the present study, the approach of using 1KGP data as the control group was feasible, since sex-related genetic differences were compared within case and control subjects, rather than between cases and controls. Additionally, we used WES data from 1000 samples from East-Asians with depressive disorder, linked to highly curated clinical data from structured diagnostic interviews and well-validated measurements. Since the majority of available resources on depression have been developed based on information collected predominantly from individuals of European ancestry, our data will help to reduce the generalizability biases arising from the under-representation of non-European populations.

### Sex-specific genetic heterogeneity in depression

Based on our qualitative analyses, we found five variants in *PDE4A*, *FDX1L*, and *MYO15B*, associated with increased risk for depressive disorder in women, that did not differ significantly between the sexes in controls. Patients homozygous for these variations, suffered from more severe depressive symptoms and higher rates of suicidality, suggesting that these five variants have clinical implications, as patients with more variant alleles experienced more severe depressive symptoms. These findings are consistent with those of previous studies that reported increased genetic liability in patients with severe forms of depression^47,48^.

No male-specific risk variants were identified by qualitative analysis. In line with these findings, previous genetic studies have reported more female-specific than male-specific genetic factors associated with depressive disorders^6–10.40,49^. This could be attributable to the unbalanced sizes of the sample populations between the sexes (70.7% females versus 29.3% males), although this reflects the epidemiological finding of a female preponderance in depressive disorders^2,3^. Additionally, it is hypothesized that males are genetically predispose to be protected against depression, which may have contributed to the lack of identification of male-specific variants in previous studies, because large genetic burden, rather than low-impact single variants, is required for the development of depressive disorders in males (see further explanation in ‘Male protective effects in depression’ below).

Interestingly, three of the five variants identified in this study mapped to chromosome 19p13.2, which is associated with a microdeletion disorder resulting in neurodevelopmental syndromes, including intellectual disability or autism spectrum disorder^50^. Altered variants in this region are also associated with physical disorders, including autoimmune (systemic lupus erythematosus)^51^, endocrine (polycystic ovary syndrome)^52^, and psychiatric disorders, such as panic disorder^53^ and schizophrenia^54^. These conditions have high rates of comorbidity with depression^55–57^ and show a preponderance of prevalence in females, except schizophrenia, which is thought to have a different clinical course, according to sex^56^. Previous familial studies of recurrent, early onset major depressive disorder showed that genes at chromosome 19p13 interact with CREB1 to increase the risk of depression^7^. Based on these previous findings, variants on chromosome 19p13.2 are good candidates for sex-specific increased risk of depressive disorder.

PDE4A is a major regulator of cAMP second messenger signaling^58^, which is considered a potential target for depression, given its wide expression in brain regions that regulate memory and mood, such as the prefrontal cortex, hippocampus, and amygdala^59^. Moreover, anxiogenic behavior and impaired emotional memory are associated with increased urine corticosterone in PDE4A-deficient mice^60^, while chronic administration of antidepressants increases PDE4A expression^61,62^. Thus, this gene may be involved in synaptic plasticity affected by antidepressants, and variants in PDE4A might decrease neuronal firing and dysregulate negative feedback via the hypothalamus-pituitary-adrenal axis, which predisposes individuals to depressive disorder. The female-specific nature of the *PDE4A* association is consistent with previous findings of high levels of PDE4 enzyme expression in the ovaries and their role in modulating steroidogenesis and inflammatory responses^63^. FDX1L (also known as FDX2) can contribute to mitochondrial myopathy and/or neurological symptoms^64,65^; however, no previous study has found associations of this factor with depressive disorder. Nevertheless, the two variants in *FDX1L* are located downstream of Ribonucleoprotein, PTB Binding 1 (RAVER1), which was identified as associated with depressive disorder, despite possible alternative interpretations of type 1 error, being in LD with another important gene, or having specific effects on gene function^66^. Variants in FDX2 and/or RAVER1 are involved in mitochondrial dysfunction, which can contribute to depression pathogenesis by resulting in oxidative stress and acceleration of apoptosis, associated neurotransmitter release^67^, and thus increased stress hormone levels^68^, particularly in a sex-specific manner^69^. Little is known about the role of *MYO15B*, which maps to chromosome 17q25.1; however, a relationship between this region and white matter hyperintensities associated with increased risk of cognitive dysfunction, dementia, and depression has been reported^70^. Further investigations are needed to fully interpret the associations with depressive disorder and their sex-specific traits discovered in this study.

### Male protective effects in depression

In our qualitative analyses, we defined various variant subcategories and used these to compare the genetic burden between males and females. Given the heterogeneity of depressive disorders, it is essential to evaluate the cumulative effects of multiple variants on depressive disorders by collapsing both common and rare variants, rather than simply focusing on classical single variant-based association tests. Among the eight annotation categories, only deleterious PTVs in autosomal protein-coding genes were associated with a significantly higher genetic burden in males than females, which is logical, as PTVs are predicted to truncate gene-coding sequences and have high impact on the genetic architectures of common disease^71,72^; however, this signal was not detected among the known depression genes in the MK4MDD database, which may be due to limitations in the generalizability and reliability of this depression-associated gene list. Further analysis of PRSs based on the recent PGC-MDD summary statistics^45^ of the European population using our WES data also supported protection against depression in males.

In contrast to our findings supporting quantitative sex differences in the genetic influence on depressive disorders, previous studies have reported no detectable differences, or inconsistent findings, in genetic effects between the sexes^11,18,19,73,74^. These discrepancies could be due to variations in analysis methods (twin studies) and ethnicity, and technical limitations for the detection of rare variants using GWAS. Moreover, limited numbers of samples subjected to exome sequencing and a lack of investigation of various annotation categories has hampered the generation of definite conclusions to date. Based on our findings, a prerequisite for a higher genetic burden for depressive disorders to develop in men could contribute to resilience against the development of depressive disorder, providing a possible biological explanation for the higher prevalence of these conditions in women.

### Limitations

Interpretation of our findings requires consideration of several limitations. First, a limited number of well-defined depression-free controls (severely injured patients with no psychiatric disorders, n = 72) were available in the present analyses. To compensate for this limitation, we used publicly available data (n = 207 from the 1KGP CHB and JPT populations) from a healthy population with similar ethnicity to our depressive subjects. Nevertheless, future investigations including larger sample sizes for both cases and controls are needed to support our findings and provide sufficient statistical power. Second, the WES findings need to be replicated in an additional large-scale depression sample with more ethnic groups; however, this is the first study to investigate sex-specific genetic risk factors for depressive disorders and could serve as a foundation for future replication studies. Third, variants outside protein-coding regions were not included; thus future research on variant subcategories with refined annotations should be tested in a sex-specific manner, including variants outside of protein-coding regions.

## Conclusions

The present study used WES data to provide strong support for the contribution of genetic variation to sex differences in depressive patients. Our findings, identifying female-specific variants for depressive disorder and a higher genetic burden prerequisite for depressive disorder onset in men, provide evidence of the biological mechanisms underlying the female preponderance in depression. Based on these data, future studies on appropriate preventive and treatment strategies should be conducted for patients with sex-specific genetic risk factors for depressive disorders.

## Supplementary information

Supplementary information.

Supplementary information 2.
