# Supplementary material for: Sex differences in the genetic architecture of depression
Source: Sci Rep. 2020 Jun 18;10:9927. doi: 10.1038/s41598-020-66672-9 (PMC7303215; doi:10.1038/s41598-020-66672-9)
Supplement: Supplementary file 1 — Supplementary information. [file 41598_2020_66672_MOESM1_ESM.docx]

**Sex differences in the genetic architecture of depression**

Hee-Ju Kang^1#,^ Yoomi Park^2#^, Kyung-Hun Yoo^2^, Ki-Tae Kim^2^, Eun-Song Kim^1^, Ju-Wan Kim^1^, Sung-Wan Kim, ^1^ Il-Seon Shin^1^, Jin-Sang Yoon^1^, Ju Han Kim^2^*^#^, Jae-Min Kim^1^*^#^

***Supplementary materials***

**Supplementary methods…………………………………………………………………..2**

Eligibility Criteria ……………………………………………………………………………………………………2

Whole exome sequencing …………………………………………………………………………………………3

References of supplementary methods………………………………………………………………………..5

**Supplementary Figure S1….……..……………………………………………………….7**

**Supplementary Figure S2.…………………………………….….………………………..8**

**Supplementary Figure S3.…………………………………….….………………………..9**

**Supplementary Figure S4.…………………………………….….………………………..10**

**Supplementary Table S1….……..…………………… ………….………………………..12**

**Supplementary Table S2.……………….…………………...……………………………..13**

**Supplementary Table S3.……………….…………………...……………………………..14**

**Supplementary Table S4.……………….…………………...…………………………......15 Supplementary Table S5.……………….…………………...……………………………..16**

**Supplementary methods**

***Eligibility criteria***

For the MAKE BETTER study, inclusion criteria were as follows: i) aged older than 7 years; ii) diagnosed with major depressive disorder, dysthymic disorder, or depressive disorder not otherwise specified (NOS), as ascertained using the Mini-International Neuropsychiatric Interview^1^; iii) Hamilton Depression Rating Scale^2^ score ≥ 14; iv) able to complete questionnaires, understand the objective of the study, and sign the informed consent form. Exclusion criteria were as follows: i) unstable or uncontrolled medical condition; ii) unable to complete the psychiatric assessment or comply with the medication regimen, due to a severe physical illness; iii) current or lifetime DSM-IV diagnosis of bipolar disorder, schizophrenia, schizoaffective disorder, schizophreniform disorder, psychotic disorder NOS, or other psychotic disorder; iv) history of organic psychosis, epilepsy, or seizure disorder; v) history of anticonvulsant treatment; vi) hospitalization for any psychiatric diagnosis except depressive disorder (e.g., alcohol/drug dependence); vii) electroconvulsive therapy for the current depressive episode; viii) pregnant or breastfeeding.

For the BioPTS study, inclusion criteria were as follows: i) hospital admission for more than 24 h after a severe physical injury (injured severity score ≥ 9)^3^; ii) > 18 years old; iii) a native Korean speaker. To maintain a representative sample, the following exclusion criteria were applied: i) moderate or severe brain injury (Glasgow coma scale < 10)^4^; ii) primary clinical diagnosis of a psychiatric disorder not associated with post-traumatic stress that could affect the course of post-traumatic stress or a diagnosis of a current psychotic or bipolar disorder, or a history of suicide attempts; iii) significant pre-existing cognitive impairments, such as organic mental problems or neurocognitive disorders.

***Whole exome sequencing***

WES was performed to screen coding sequence regions across the entire genome using the Illumina HiSeq 2500 sequencer (Illumina, Inc., San Diego, CA), with standard protocols, as described in the manufacturer’s instructions. The SureSelect Human All Exon V5+UTR probe set, which includes 359,555 exons of 21,522 genes and has a total targeted region of 75 Mb, was used. To generate standard exome capture libraries, the Agilent SureSelect Target Enrichment protocol for Illumina paired-end sequencing libraries (ver. B.3, June 2015) was used, with 3 µg of input gDNA. DNA quantity and quality were measured using PicoGreen reagent and a Nanodrop spectrophotometer. Genomic DNA aliquots (1 µg) were fragmented using adaptive focused acoustic technology (Covaris). Fragmented DNA was repaired, an ‘A’ residue was ligated to the 3′ end, and Agilent adapters were then ligated to the fragments. Once ligation had been assessed, adapter ligated products were amplified by PCR and the final purified products were quantified using qPCR, based on the qPCR Quantification Protocol Guide, and quality was assessed using the Caliper LabChip High Sensitivity DNA kit (PerkinElmer, Inc. Hopkinton, MA). For exome capture, 250 ng of DNA library was mixed with hybridization buffer, blocking mix, RNase block, and 5 µl of SureSelect all exon capture library, according to the standard Agilent SureSelect Target Enrichment protocol. Hybridization to capture baits was conducted at 65°C using a heated thermal cycler lid option at 105°C for 24 h on a PCR machine. The captured DNA was then amplified, and the final purified products were quantified by qPCR using the qPCR Quantification Protocol Guide and quality assessed using TapeStation DNA screen tape (Agilent). Next, sequencing was conducted on the HiSeq™ 2500 platform (Illumina, Inc., San Diego, CA). Reads were mapped to the human genome reference sequence (hg19/GRCh37) using BWA-MEM (v0.7.7) ^5,6^. Aligned reads were sorted and indexed using SAMTools(v0.1.19)^7^, and PCR duplicates were marked using MarkDuplicates in the Picard toolkit (http://broadinstitute.github.io/picard). The Genome Analysis Toolkit (GATK, v2.8.1)^8-10^was used for local realignment and base recalibration (dbSNP137 for single nucleotide variants(SNVs), and Mills and 1000 Genome Project gold-standard InDels from hg19 sites for Indels). SNVs and short insertions/deletions (InDels) were identified using HaplotypeCaller in GATK. Variants were hard-filtered, according to the recommendations listed in the Best Practice Variant Detection documentation in GATK with the following parameters: “QD < 2.0”, “MQ < 40.0”, “FS > 60.0”, “MQRankSum < 12.5”, “ReadPosRankSum < −8.0” for SNPs; and “QD < 2.0”, “ReadPosRankSum < −20.0”, “FS > 200.0” for InDels. To reduce the likelihood of false-positive calls, variants assigned as SnpCluster (clusterSize = 3, clusterWindowSize = 35) by GATK were filtered out. Protein-coding gene regions were defined using SnpEff 4.1 (build 2015-01-07)^11^Variants with putative functional impact were chosen based on the following variant types: missense, stop gained, stop lost, and start lost. To estimate the pathogenicity of variants, data were annotated using three *in silico* variant deleteriousness prediction scores: Sorting Intolerant From Tolerant (SIFT)^12^, PolyPhen2 (PP2)^13^, and Combined Annotation Dependent Depletion (CADD)^14^. For resolution comparable to that of WES data, only variants in the same target region were extracted from the 1KGP whole genome sequencing data and used in downstream analyses.

**Reference of Supplementary Methods**

1. Sheehan, D.V., et al. The mini-international neuropsychiatric interview (M.I.N.I): the development and validation of a structured diagnostic psychiatric interview for DSM-IV and ICD-10. *J. Clin. Psychiatry* **59**, S22-S33 (1998).
2. Hamilton, M. A rating scale for depression. *J. Neurol.Neurosurg. Psychiatry* **23**, 56-62 (1960).
3. Baker, S.P., O’Neil, B., Haddon, W. Jr. & Long, W.B. The injury severity score: a method for describing patients with multiple injuries and evaluating emergency care. *J. Trauma* **14**, 187-196 (1974).
4. Teasdale, G. & Jennett, B. Assessment of coma and impaired consciousness. A practical scale. *Lancet* **2**, 81-84 (1974).
5. Li, H. & Durbin, R. Fast and accurate short read alignment with Burrows-Wheeler transform. *Bioinformatics* **25**, 1754–1760 (2009).
6. Li,,H. & Durbin,,R. Fast and accurate long-read alignment with Burrows-Wheeler transform. *Bioinformatics* **26**, 589–595 (2010).
7. Li, H., et al. The Sequece Alignment/Map format and SAM tools. *Bioinformatics* **25**, 2078-2079 (2009).
8. McKenna, A., et al. The Genome Analysis Toolkit: A MapReduce framework for analyzing next-generation DNA sequencing data. *Genome Res.* **20**, 1297–1303 (2010).
9. DePristo, M.A., et al. A framework for variation discovery and genotyping using next-generation DNA sequencing data. *Nat. Genet.* **43**, 491–498 (2011).
10. Van der Auwera, G.A., et al. From FastQ data to high confidence variant calls: the Genome Analysis Toolkit best practices pipeline. *Curr. Protoc. Bioinformatics* **43**, 11.10.1-11.10.33 (2013).
11. Cingolani, P., et al. A program for annotating and predicting the effects of single nucleotide polymorphisms, SnpEff: SNPs in the genome of Drosophila melanogaster strain w1118; iso-2; iso-3. *Fly (Austin)* **6**, 80-92 (2012).
12. Ng, P.C. & Henikoff, S. SIFT: predicting amino acid changes that affect protein function. *Nucleic Acids Res.* **31**, 3812–3814 (2003).
13. Adzhubei, I.A., et al. A method and server for predicting damaging missense mutations. *Nat. Methods* **7**, 248-249 (2010).
14. Kircher, M., Witten, D.M., Jain, P., O'Roak, B.J., Cooper, G.M. & Shendure, J. A general framework for estimating the relative pathogenicity of human genetic variants. *Nat. Genet.* **46**, 310–315 (2014).

**Supplementary Figure S1. Schematic data analysis steps**

Five *in silico* tools were used to select predicted deleterious variants: SIFT, PolyPhen2, CADD, LRT, and MutationTaster.

SIFT= Sorting Intolerant From Tolerant; CADD= Combined Annotation Dependent Depletion; LRT=Likelihood Ratio Test; MDD=Major Depressive Disorder; VCF=Variant Call Format; 1KGP = 1000 Genomes Project; CHBJPT = Han Chinese in Beijing, China (CHB) data and Japanese in Tokyo, Japan (JPT) data from the 1000 Genomes Project.


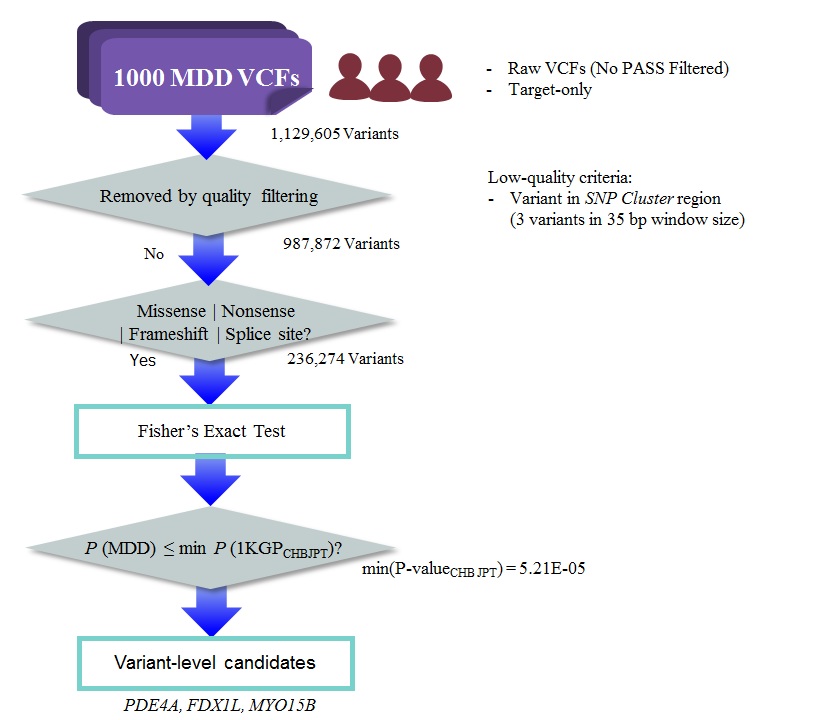


**Supplementary Figure S2. Outline of permutation testing**


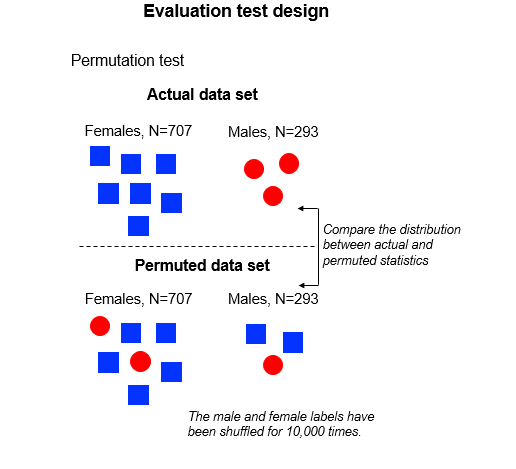


**Supplementary Figure S3. Comparison of the genetic burden with modified rare variant definition (allele frequency < 1%) between men and women in patients with depressive disorder and the general population.**

Comparison of genetic variants in each category (points) enriched in males (relative genetic burden > 1) or females (relative genetic burden < 1). The red dashed line indicates the significance threshold (0.05). (a) Patients with depressive disorder (n = 1000). (b) Healthy controls from the 1KGP CHB and JPT populations (n = 207).

1KGP = 1000 Genomes Project; CHBJPT = Han Chinese in Beijing, China (CHB) data and Japanese in Tokyo, Japan (JPT) data from the 1000 Genomes Project.


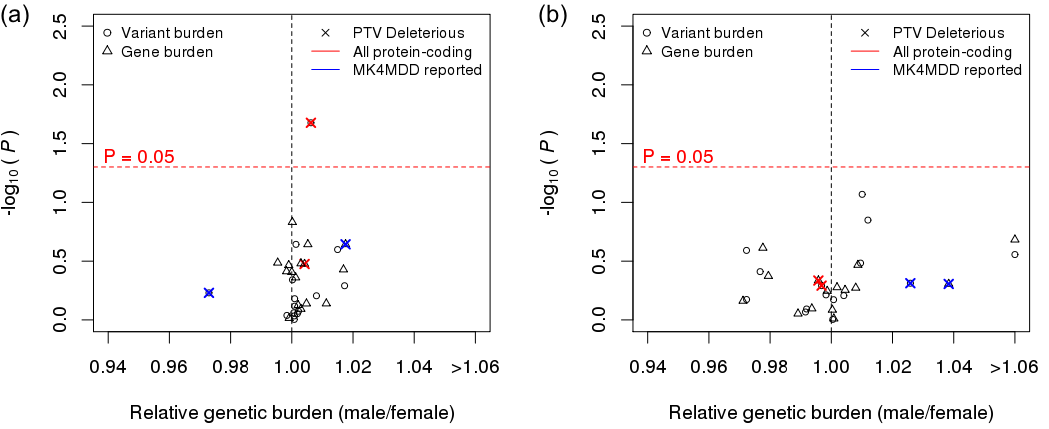


**Supplementary Figure S4. Comparison of polygenic risk scores (PRSs) between men and women in patients with depressive disorders and the general population.**

PRSs were obtained using LD-clumped independent SNPs with p-values for association below eight thresholds (P< 10^−4^, 0.001, 0.01, 0.05, 0.1, 0.2, 0.5 and 1). The figures in the upper row represent the distribution of PRSs in patients with depressive disorder (n = 1000) while the figures in the lower row represent distribution of PRSs in healthy controls from the Biomarker-Based Diagnostic Algorithm for Posttraumatic Syndrome study (n = 72).


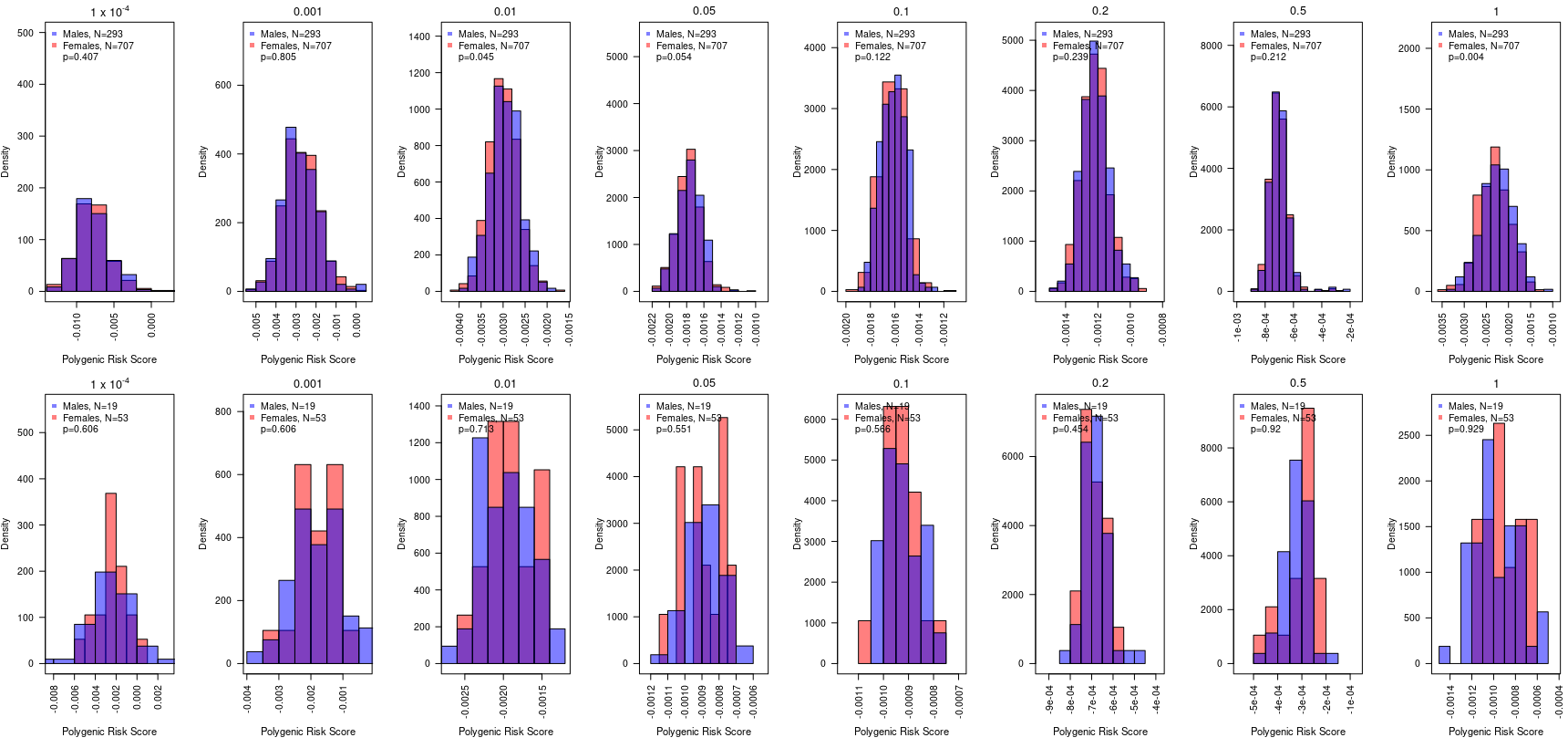


| **Supplementary Table S1 Definition of genetic burden** | |
| --- | --- |
| Group | Description |
| Rare | 1KGP MAF < 0.1% |
| Functional | Missense, splice site, frameshift, stop lost, stop gain, stop retained, start lost, inframe InDels |
| Protein Truncating Variants (PTVs) | Splice site, frameshift, stop lost, stop gain, stop retained, start lost |
| Deleterious | SIFT ≤ 0.05 or CADD ≥ 15 |
| 1KGP, 1000 Genomes Project, EAS, East Asian population, AF, Allele Frequency | |

| **Supplementary Table S2 Description of the study group** | | | | |
| --- | --- | --- | --- | --- |
| Phenotype | Ethnicity | Samples | Females | Males |
| Major Depressive Disorder | Korean | 1000 | 707 | 293 |
| Control from BioPTS | Korean | 72 | 19 | 53 |
| (No psychiatric disorders even after severe physical injury) | |  |  |  |
| Control from general population  (healthy normal) | Han Chinese in Bejing, China (CHB) and Japanese in Tokyo, Japan (JPT) | 207 | 105 | 102 |
|  | All the mixed population of the 1000 Genomes Project (1KGP) | 2504 | 1271 | 1233 |
| BioPTS, Biomarker-Based Diagnostic Algorithm for posttraumatic syndrome after physical injury | | | | |

**Supplementary Table S3** Allele and genotype frequencies of potential candidate genes between males and females (P < 1E-03)

Provide as a separate XLSX file (See Supplementary file 2)

| **Supplementary Table S4 Independent sex-specific associations of genetic variants on depressive disorders** | | | | | | | | | | | | | |
| --- | --- | --- | --- | --- | --- | --- | --- | --- | --- | --- | --- | --- | --- |
| Gene | Variant | SIFT | CADD | PP2 | Exon | HGVS.p | Type | Multi-variate logistic regression test | | |  | Cochran-Mantel-Haenszel test | |
|  |  |  |  |  |  |  |  | Statistical  coeffients (β) | OR  (95% CI) | p-value |  | Statistical  coeffients (𝜒2) | p-value |
| *PDE4A* | rs201432982 | 0.04 | 23.5 | NA | 1/15 | p.Arg57Trp | Missense | 0.962 | 2.62 (1.6-4.4) | 1.74E-04 |  | 27.82 | 0.023 |
| *FDX1L*  *(RAVER1* downstream*)* | rs62640397 | 0.00 | 0.017 | 0.069 | 1/5 | p.Arg29Gly | Missense | 0.850 | 2.34 (1.5-3.7) | 1.31E-04 |  | 32.44 | 5.61E-03 |
|  | rs79442975 | NA | 9.108 | NA | 1/4 | NA | Splice region variant |  |  |  |  |  |  |
| *MYO15B* | rs820182 | NA | 5.116 | NA | 48/62 | NA | Splice region variant | 0.614 | 1.85 (1.4-2.5) | 8.89E-05 |  | 29.71 | 0.013 |
|  | rs820148 | NA | 6.033 | NA | 45/62 | NA | Splice region variant | 0.575 | 1.78  (1.3-2.5) | 3.87E-04 |  | 28.97 | 0.016 |
| Data were adjusted for age, education, unemployment status, diagnosis of depression, recurrent depression, family history of depression, and Hamilton Depression Rating Scale baseline score  SIFT, Sorting Intolerant From Tolerant; PP2, PolyPhen2; CADD, Combined annotation dependent depletion; HGVS.p, Human genome variation society –protein reference sequence; OR, Odds Ratio, CI, Confidence Interval, LR, Logistic Regression | | | | | | | | | | | | | |

| **Supplementary Table S5. Clinical characteristics of depressive patients with and without sex-specific haplotypes based on the five identified genetic variants.** | | | | | | | | | | | | | | |
| --- | --- | --- | --- | --- | --- | --- | --- | --- | --- | --- | --- | --- | --- | --- |
| **Variables** | **Depressive patients, N=1000** | | | | **Depressive patients, Female, N=707** | | | | | **Depressive patients, Male, N=293** | | | | |
| **PDE4A-FDX1L-MYO15B**  **(T-C-A-C-G Haplotype)** | **Carrier, N=32** | **Non-carrier, N=968** | **Statistical coefficient** | **P-value** | | **Carrier, N=31** | **Non-carrier, N=676** | **Statistical coefficient** | **P-value** | | **Carrier, N=1** | **Non-carrier, N=272** | **Statistical coefficient** | **P-value** |
| HAMD baseline score, mean (sd) | 23.66 (4.8) | 20.59 (4.2) | W=21081.5 | **4.85E-04** | | 23.77 (4.9) | 20.78 (4.2) | W=14178.5 | **8.48E-04** | | 20 (NA) | 20.16 (4.1) | W=152.5 | 0.943 |
| HADS-anxiety subscale, mean(sd) | 12.78 (3.8) | 11.73 (4) | W=18014.5 | 0.115 | | 13 (3.7) | 11.67 (4) | W=12610 | 0.055 | | 6 (NA) | 11.87 (4) | W=24.0 | 0.150 |
| BPRS suicide item≥4, n(%) | 19 (59.4) | 314 (32.4) | 𝜒2=8.94 | **2.79E-03** | | 19 (61.3) | 220 (32.5) | 𝜒2=9.7 | **1.84E-03** | | 0 (0) | 94 (32.2) | 𝜒2=0 | 1 |
| Suicidal attempt (y) | 3 (9.4) | 83 (8.6) | 𝜒2=0 | 1 | | 3 (9.7) | 52 (7.7) | 𝜒2=0 | 0.952 | | 0 (0) | 31 (10.6) | 𝜒2=0 | 1 |
| Family history (y), n(%) | 6 (18.8) | 144 (14.9) | 𝜒2=0.12 | 0.725 | | 6 (19.4) | 110 (16.3) | 𝜒2=0.04 | 0.837 | | 0 (0) | 34 (11.6) | 𝜒2=0 | 1 |
| Recurrent depression, n(%) | 16 (50) | 494 (51) | 𝜒2=0 | 1 | | 16 (51.6) | 366 (54.1) | 𝜒2=0.01 | 0.927 | | 0 (0) | 128 (43.8) | 𝜒2=0 | 1 |
| Age of onset, mean(sd) | 51.03 (15.3) | 51.99(16.5) | W=14688 | 0.619 | | 50.52(15.3) | 51.62 (15.9) | W=9908.5 | 0.609 | | 67 (NA) | 52.86 (17.7) | W=221.0 | 0.378 |
| **PDE4A-FDX1L**  **(T-C-A Haplotype)** | **Carrier, N=10** | **Non-carrier, N=990** | **Statistical coefficient** | **P-value** | | **Carrier, N=10** | **Non-carrier, N=697** | **Statistical coefficient** | **P-value** | | **Carrier, N=0** | **Non-carrier, N=293** | **Statistical coefficient** | **P-value** |
| HAMD baseline score, mean (sd) | 23.3 (4.0) | 20.66 (4.2) | W=6765 | 0.045 | | 23.3 (4.0) | 20.88 (4.3) | W=4673 | 0.063 | | NA | 20.16 (4.1) | NA | NA |
| HADS-anxiety subscale, mean(sd) | 12.4 (4.6) | 11.76 (4.0) | W=5491 | 0.551 | | 12.4 (4.6) | 11.71 (4.0) | W=3880 | 0.537 | | NA | 11.9 (4.0) | NA | NA |
| BPRS suicide item≥4, n(%) | 6 (60.0) | 327 (33.0) | 𝜒2=2.14 | 0.143 | | 6 (60.0) | 233 (33.4) | 𝜒2=2.04 | 0.154 | | 0(0) | 94 (32.1) | 𝜒2=0 | 1 |
| Suicidal attempt (y) | 1 (10.0) | 85 (8.6) | 𝜒2=0 | 1 | | 1 (10.0) | 54 (7.7) | 𝜒2=0 | 1 | | 0(0) | 31 (10.6) | 𝜒2=0 | 1 |
| Family history (y), n(%) | 1 (10.0) | 149 (15.1) | 𝜒2=0 | 1 | | 1 (10.0) | 115 (16.5) | 𝜒2=0.01 | 0.904 | | 0(0) | 34 (11.6) | 𝜒2=0 | 1 |
| Recurrent depression, n(%) | 5 (50.0) | 505 (51.0) | 𝜒2=0 | 1 | | 5 (50.0) | 377 (54.1) | 𝜒2=0 | 1 | | 0(0) | 128 (43.7) | 𝜒2=0 | 1 |
| Age of onset, mean(sd) | 52.6 (15.8) | 51.96 (16.4) | W=4845 | 0.908 | | 52.6 (15.8) | 51.56 (15.9) | W=3465 | 0.976 | | NA | 52.9 (17.7) | NA | NA |
| **MYO15B**  **(C-G Haplotype)** | **Carrier, N=22** | **Non-carrier, N=978** | **Statistical coefficient** | **P-value** | | **Carrier, N=21** | **Non-carrier, N=686** | **Statistical coefficient** | **P-value** | | **Carrier, N=1** | **Non-carrier, N=292** | **Statistical coefficient** | **P-value** |
| HAMD baseline Score, mean (sd) | 23.82 (5.3) | 20.62 (4.2) | W=14536.5 | **0.005** | | 24 (5.3) | 20.82 (4.2) | W=9715.5 | **0.006** | | 20 (NA) | 20.16 (4.1) | W=152.5 | 0.943 |
| HADS-anxiety subscale, mean(sd) | 12.95 (3.5) | 11.74 (4.0) | W=12743.5 | 0.137 | | 13.29 (3.3) | 11.68 (4.1) | W=8940 | 0.059 | | 6 (NA) | 11.87 (4) | W=24.0 | 0.150 |
| BPRS suicide item≥4, n(%) | 13 (59.1) | 320 (32.7) | 𝜒2=5.6 | 0.018 | | 13 (61.9) | 226 (32.9) | 𝜒2=6.4 | 0.011 | | 0 (0) | 94 (32.2) | 𝜒2=0 | 1 |
| Suicidal attempt (y) | 2 (9.1) | 84 (8.6) | 𝜒2=0 | 1 | | 2 (9.5) | 53 (7.7) | 𝜒2=0 | 1 | | 0 (0) | 31 (10.6) | 𝜒2=0 | 1 |
| Family history (y), n(%) | 5 (22.7) | 145 (14.8) | 𝜒2=0.52 | 0.469 | | 5 (23.8) | 111 (16.2) | 𝜒2=0.4 | 0.528 | | 0 (0) | 34 (11.6) | 𝜒2=0 | 1 |
| Recurrent depression, n(%) | 11 (50.0) | 499 (51.0) | 𝜒2=0 | 1 | | 11 (52.4) | 371 (54.1) | 𝜒2=0 | 1 | | 0 (0) | 128 (43.8) | 𝜒2=0 | 1 |
| Age of onset, mean(sd) | 50.32 (15.4) | 52 (16.4) | W=10063 | 0.604 | | 49.52(15.4) | 51.63 (15.9) | W=6653.5 | 0.551 | | 67 (NA) | 52.86 (17.7) | W=221.0 | 0.378 |
| P-Values were analyzed using the Wilcoxon rank-sum or chi-squared test, as appropriate.  Values in bold type show statistical significance after Bonferroni correction.  HAMD, Hamilton Depression Rating Scale; HADS, Hospital Anxiety Depression Scale; BPRS, Brief Psychiatric Rating Scale; NA, not applicable | | | | | | | | | | | | | | |
